# Cell-Specific IRF-3 Responses Protect against West Nile Virus Infection by Interferon-Dependent and -Independent Mechanisms

**DOI:** 10.1371/journal.ppat.0030106

**Published:** 2007-07-27

**Authors:** Stephane Daffis, Melanie A Samuel, Brian C Keller, Michael Gale, Michael S Diamond

**Affiliations:** 1 Department of Medicine, Washington University School of Medicine, St. Louis. Missouri, United States of America; 2 Department of Molecular Microbiology, Washington University School of Medicine, St. Louis. Missouri, United States of America; 3 Department of Microbiology, University of Texas Southwestern Medical Center, Dallas, Texas, United States of America; 4 Department of Pathology and Immunology, Washington University School of Medicine, St. Louis, Missouri, United States of America; Cleveland Clinic, United States of America

## Abstract

Interferon regulatory factor (IRF)-3 is a master transcription factor that activates host antiviral defense programs. Although cell culture studies suggest that IRF-3 promotes antiviral control by inducing interferon (IFN)-β, near normal levels of IFN-α and IFN-β were observed in IRF-3^−/−^ mice after infection by several RNA and DNA viruses. Thus, the specific mechanisms by which IRF-3 modulates viral infection remain controversial. Some of this disparity could reflect direct IRF-3-dependent antiviral responses in specific cell types to control infection. To address this and determine how IRF-3 coordinates an antiviral response, we infected IRF-3^−/−^ mice and two primary cells relevant for West Nile virus (WNV) pathogenesis, macrophages and cortical neurons. IRF-3^−/−^ mice were uniformly vulnerable to infection and developed elevated WNV burdens in peripheral and central nervous system tissues, though peripheral IFN responses were largely normal. Whereas wild-type macrophages basally expressed key host defense molecules, including RIG-I, MDA5, ISG54, and ISG56, and restricted WNV infection, IRF-3^−/−^ macrophages lacked basal expression of these host defense genes and supported increased WNV infection and IFN-α and IFN-β production. In contrast, wild-type cortical neurons were highly permissive to WNV and did not basally express RIG-I, MDA5, ISG54, and ISG56. IRF-3^−/−^ neurons lacked induction of host defense genes and had blunted IFN-α and IFN-β production, yet exhibited only modestly increased viral titers. Collectively, our data suggest that cell-specific IRF-3 responses protect against WNV infection through both IFN-dependent and -independent programs.

## Author Summary

West Nile virus (WNV) is a mosquito-transmitted RNA virus that infects birds, horses, and humans, and it has become an emerging infectious disease threat in the Western hemisphere, including all of the continental United States. WNV invades the brain and spinal cord and infects and injures neurons, causing severe neurological disease, including encephalitis and paralysis, primarily in the immunocompromised and elderly. An increased understanding of how the immune system recognizes WNV and promotes an antiviral defense is vital to developing novel therapeutics and vaccines that limit disease, and identifying individuals at high risk for severe disease. In this study, using a mouse model of WNV pathogenesis, we evaluate the functional role of interferon regulatory factor 3 (IRF-3), a master transcriptional regulator of interferon induction and antiviral responses, in controlling infection. Mice that lack IRF-3 were uniformly susceptible to severe infection with 100% lethality. We observed cell-type–specific responses, as neurons and macrophages utilized IRF-3 to protect against WNV infection through distinct antiviral pathways. Finally, IRF-3 also appears to regulate the basal expression of specific sensors of viral infection, which in turn affects the antiviral state of a cell prior to virus entry.

## Introduction

West Nile virus (WNV) is a mosquito-borne, positive polarity, single-stranded RNA virus in the Flaviviridae family. In humans, WNV causes a spectrum of illness that ranges from a self-limiting WNV fever to flaccid paralysis and fatal encephalitis [1]. WNV is endemic in Africa, Europe, Asia, and Australia, and has emerged in the Western hemisphere with annual outbreaks in North America; additionally, WNV has recently spread to Mexico and Central and South America.

Rodent models have helped to elucidate the mechanisms of WNV dissemination and pathogenesis. Following mosquito inoculation, WNV replicates in Langerhans and other naive dendritic cells in the skin [2,3]. WNV then spreads to the draining lymph nodes and spleen, where viral amplification occurs in subsets of CD11b^+^ myeloid cells [4]. Virus subsequently disseminates to the central nervous system (CNS) and infects neurons [5,6]. Innate and adaptive immune responses are required for control and clearance of WNV infection (reviewed in [7]). Induction of interferon (IFN)-α and IFN-β genes is essential to the host response against viral infections, including WNV [8–10]. Mice lacking the IFN-α and IFN-β receptor (IFN-α/βR^−/−^) were extremely susceptible to WNV infection, with expanded viral tropism in myeloid cells, early dissemination in the CNS, uncontrolled viral replication, and early and uniform death.

Studies by several groups have begun to define the molecular mechanisms by which immune and nonimmune cells detect and respond to RNA viruses (reviewed in [11,12]). Binding of single-stranded or double-stranded viral RNA to retinoic acid–inducible gene (RIG)-I, melanoma differentiation antigen (MDA)5, Toll-like receptor (TLR)3, TLR7, or TLR8 results in downstream activation of transcription factors, such as interferon regulatory factors 3 and 7 (IRF-3 and IRF-7), production of IFN-α and IFN-β, and the expression of IFN-stimulated genes (ISGs).

An emerging literature suggests that RIG-I and IRF-3 have essential functions in the response to WNV infection. Murine embryonic fibroblasts (MEFs) deficient in RIG-I have a delayed host defense response, decreased IRF-3 activation, and augmented WNV replication [13,14]. In contrast, MDA5 and TLR3, pathogen recognition receptors that also signal through IRF-3, may be less essential for flavivirus recognition and control. No distinct in vivo phenotype was observed in MDA5^−/−^ mice after infection with the closely related flavivirus, Japanese encephalitis virus [15]. TLR3 may be dispensable for recognition of WNV in some cell types in vitro [13,16] and in vivo [17], as only small differences in viral yield were observed in the absence of TLR3.

Induction of an optimal IFN response after RNA virus infection likely requires signaling through both IRF-3 and IRF-7 [18–21]. IRF-3^−/−^ mice are more susceptible to encephalomyocarditis virus (EMCV), and IRF-3^−/−^ MEFs exhibited a reduced capacity to produce IFN after infection with several RNA and DNA viruses, including Newcastle disease virus, herpes simplex virus (HSV), vesicular stomatitis virus (VSV), and EMCV [19,22]. Nonetheless, others have reported that IRF-3 is not required for IFN production in dendritic cells after RNA virus infection (VSV, EMCV), and serum levels of IFN-α and IFN-β after HSV, EMCV, and Semliki Forest virus (SFV) infection in IRF-3^−/−^ mice did not differ substantially from those of congenic wild-type controls [22,23]. The role of IRF-3 in regulating the basal expression of host defense genes, inducing IFN, and promoting an antiviral response in different primary cell types with a given virus remains unknown.

We evaluated the function of the master transcriptional regulator IRF-3 in vivo and ex vivo in cell types relevant to WNV pathogenesis. Mice lacking IRF-3 were acutely vulnerable to WNV infection with uniform mortality. IRF-3 was essential for controlling WNV replication in peripheral tissues and myeloid cells through largely IFN-independent pathways. Moreover, in cortical neurons, IRF-3 had a smaller antiviral function, yet regulated production of IFN-α and IFN-β. These data suggest that cell-specific IRF-3 responses protect against WNV infection through both IFN-dependent and -independent mechanisms.

## Results

### IRF-3 Is Required for Controlling Lethal WNV Infection

**Figure 1:**
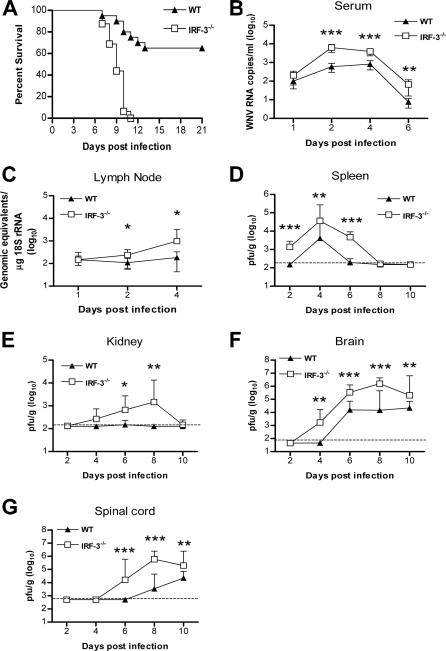
Survival and Virologic Analysis for Wild-Type and IRF-3^−/−^ C57BL/6 Mice (A) Eight- to twelve-week-old mice were inoculated with 10^2^ PFU of WNV by footpad injection and followed for mortality for 21 d. Survival differences were statistically significant (*n* = 20, IRF-3^−/−^; and *n* = 20, wild-type mice; *p* < 0.0001). (B–G) Viral burden in peripheral and CNS tissues after WNV infection. WNV RNA in (B) serum and (C) draining lymph node, and infectious virus in the (D) spleen, (E) kidney, (F) brain, and (G) spinal cord were determined from samples harvested on days 1, 2, 4, 6, 8, and 10 using qRT-PCR (B, C) or viral plaque assay (D–G). Data is shown as viral RNA equivalents or PFU per gram of tissue for ten to 12 mice per time point. For all viral load data, the solid line represents the median PFU per gram at the indicated time point, and the dotted line represents the limit of sensitivity of the assay. Error bars indicate the standard deviations (SD). Asterisks indicate values that are statistically significant (*, *p* < 0.05; **, *p* < 0.005; ***, *p* < 0.0001) compared to wild-type mice.

Mice lacking IFN-α and IFN-β receptors are extremely vulnerable to WNV infection due to rapid and overwhelming viral replication [9,24]. To dissect the mechanisms regulating IFN induction after WNV infection in vivo, we evaluated the effect of a genetic deficiency of IRF-3, a known regulator of IFN induction, on survival. After footpad inoculation with 10^2^ PFU of WNV, IRF-3^−/−^ mice showed an increased rate and severity of acute clinical symptoms, including hunchback posture, weight loss, fur ruffling, and reduced activity. IRF-3^−/−^ mice were significantly more vulnerable to lethal WNV infection, with a 0% survival rate and a mean time to death of 9.3 ± 1.1 days compared to wild-type mice, which had a 65% survival rate and a mean time to death of 10.7 ± 1.6 (*p* < 0.0001, Figure 1A). Thus, signaling through IRF-3 is essential for protecting mice against lethal WNV infection.

### IRF-3^−/−^ Mice Have Enhanced WNV Replication

Because IFN has an essential role in controlling WNV dissemination, we hypothesized that a deficiency of IRF-3 would result in higher tissue viral burdens. To evaluate this, IRF-3^−/−^ and wild-type mice were infected with 10^2^ PFU of WNV via a footpad route, and viral burden was measured by fluorogenic quantitative reverse transcriptase polymerase chain reaction (qRT-PCR) or viral plaque assay at days 1, 2, 4, 6, 8, and 10 post infection in blood, peripheral organs (draining lymph nodes, spleen, and kidney), and the CNS (brain and spinal cord).

#### Blood and lymph node.

In the absence of IRF-3, significantly higher levels of viral RNA were observed by days 2 and 4 after infection in serum (10.6-fold, *p* < 0.0001; and 4.8-fold, *p* < 0.0001; Figure 1B) and draining popliteal lymph nodes (2.2-fold, *p* = 0.03; and 5.3-fold, *p* = 0.02; Figure 1C). At day 6, viremia in wild-type mice declined, whereas WNV RNA levels remained elevated in IRF-3^−/−^ mice (*p* < 0.005).

#### Spleen and kidney.

In wild-type mice, infectious virus was not detected in the spleen by plaque assay at day 2 after infection (*n* = 10). In contrast, 100% (10 of 10) of IRF-3^−/−^ mice had measurable infection at day 2 (average titer 10^3.1^ PFU/g, *p* < 0.0001) (Figure 1D). Increased WNV was also observed in IRF-3^−/−^ mice at day 4 (average titer 10^4.6^ PFU/g versus 10^3.6^ PFU/g for wild type, *p* = 0.003), which corresponded to the peak of infection in both groups. High levels of infectious WNV (average titer 10^3.7^ PFU/g, *p* < 0.0001) were still detectable at day 6 in all (10 of 10) IRF-3^−/−^ mice. In contrast, WNV was cleared from the spleen in the majority (7 of 10) of wild-type mice at this time, with remaining animals having lower viral burdens. By day 8, WNV was largely cleared from the spleen of IRF-3^−/−^ and wild-type mice.

Little or no replication occurs in the kidney after peripheral WNV infection of wild-type C57BL/6 mice. In contrast, ∼10^6^ PFU/g of WNV was observed in the kidneys of IFN-α/βR^−/−^ mice [9], which indicates a role for IFN in restricting tissue tropism. Significant WNV infection was also observed in the kidney of IRF-3^−/−^ mice (day 6, 10^2.8^ PFU/g, *p* < 0.05; day 8, 10^3.2^ PFU/g, *p* < 0.005; Figure 1E). Based on virologic data from the serum, lymph node, spleen, and kidney, IRF-3 signaling modulates WNV infection in peripheral tissues and functions to restrict tissue tropism.

#### CNS.

WNV spread more rapidly and replicated to higher levels in the CNS of IRF-3^−/−^ mice. Infectious WNV was present in the brains of IRF-3^−/−^ mice (9 of 10) at day 4 after infection, whereas in wild-type mice, WNV was not detected until day 6. IRF-3^−/−^ mice also averaged significantly higher viral titers than wild-type mice in the brain on days 6, 8, and 10 after infection (20-fold, *p* < 0.0001; ∼200-fold, *p* < 0.0001; and 10-fold, *p* < 0.005, respectively), which corresponded with increased morbidity and mortality (Figure 1F). A similar pattern was observed in the spinal cord, where earlier entry was observed in IRF-3^−/−^ mice with 60% (6 of 10) having detectable viral loads at day 6 in contrast to 0% (0 of 10) of wild-type mice. Moreover, higher viral burden was also observed in the spinal cord of IRF-3^−/−^ mice at days 8 and 10, with ∼220-fold (*p* < 0.0001) and ∼10-fold (*p* < 0.005) increased titers compared to wild-type mice, respectively (Figure 1G). Overall, an absence of IRF-3 signaling led to increased WNV infection in peripheral tissues, resulting in early spread and increased replication in the CNS.

### Production of WNV-Specific IgM and IgG Antibodies in IRF-3^−/−^ Mice

IFN-α and IFN-β are immunomodulatory cytokines with specific roles in priming adaptive immune responses [25]. Because a higher viremia was observed in mice that lacked IRF-3, we reasoned that this could be due to depressed WNV-specific antibody responses. Interestingly, higher rather than lower titers of WNV-specific IgM and IgG were detected in IRF-3^−/−^ mice (unpublished data), possibly secondary to the increased viral burden in the lymph node and spleen. Because WNV-specific antibody responses were not blunted in IRF-3^−/−^ mice, it is likely that the increased infection in the periphery was not due to inadequate priming of B cell responses.

### IRF-3 Has a Small and Transient Effect on IFN-α and IFN-β Gene Induction in Draining Lymph Nodes

**Figure 2:**
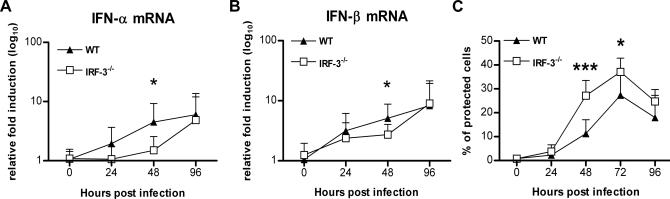
IFN Induction in Draining Lymph Nodes and Serum of Mice Infected with WNV (A, B) Mice were inoculated with 10^2^ PFU of WNV by footpad injection and euthanized at the indicated times. Total RNA from draining lymph was analyzed for (A) IFN-α and (B) IFN-β mRNA expression by qRT-PCR. Data are normalized to 18S rRNA and are expressed as the relative fold increase over normalized RNA from uninfected controls. Average values are from five to 12 mice per time point, and error bars indicate the SD. Asterisks indicate differences that are statistically significant (*, *p* < 0.05). (C) IFN activity was determined from serum collected on days 1 to 4 after infection by an EMCV bioassay in L929 cells. Data reflect the average of serum samples harvested from five to ten mice per time point and are shown as the percentage of cells protected from lysis by EMCV (see Materials and Methods). Asterisks indicate differences that are statistically significant (*, *p* < 0.05; ***, *p* < 0.0001).

In vitro studies suggest that recognition of double-stranded RNA viruses by RIG-I, MDA5, and/or TLR3 results in IRF-3-dependent transcription of IFN-β and other ISGs [15,26,27]. Nonetheless, studies in mice with EMCV, SFV, and HSV have shown that an absence of IRF-3 does not substantially alter systemic production of IFN-α and IFN-β, presumably because of signaling redundancies. To directly test the role of IRF-3 on IFN induction soon after WNV infection in vivo, we measured levels of IFN-α and IFN-β mRNAs in the draining lymph nodes of WNV-infected mice. In wild-type mice, IFN-α and IFN-β mRNA levels were induced within 24 h of WNV infection (∼2-fold increase for IFN-α and 3-fold increase for IFN-β). By 48 h and 96 h post infection, IFN-α and IFN-β tissue mRNA levels increased ∼5-fold and ∼7-fold, respectively (Figure 2A). In IRF-3^−/−^ mice, the induction of the IFN-α and IFN-β genes at 24 h was comparable to levels found in wild-type mice. However, at 48 h, there was a small yet statistically significant decrease in IFN gene induction in the draining lymph nodes of IRF-3^−/−^ mice compared to that of wild-type mice (IFN-α, *n* = 15, *p* = 0.009; IFN-β, *n* = 12, *p* = 0.05; Figure 2B). By 96 h after infection, the induction of IFN-α and IFN-β genes was similar to that observed in wild-type mice. Thus, a deficiency of IRF-3 in vivo had a small and transient effect on early IFN gene induction in lymphoid tissues after infection with WNV.

### Systemic Levels of IFN-α and IFN-β Are Similar in IRF-3^−/−^ and Wild-Type Mice

As the absence of IRF-3 partially modulated IFN gene induction in the lymph node, we hypothesized that the virologic phenotype observed in IRF-3^−/−^ mice could be due to depressed levels of IFN-α and IFN-β in circulation. To evaluate this, IRF-3^−/−^ and wild-type mice were infected with WNV, and the presence of biologically active IFN in serum was monitored using a previously validated EMCV-L929 cell bioassay [4,28]. IFN activity in the serum of infected wild-type mice was detected at 24 h, peaked at 72 h, and then decreased at 96 h (Figure 2C). Somewhat surprisingly, IFN activity in the WNV-infected IRF-3^−/−^ mice was equivalent to or greater than that observed in wild-type mice. For example, 2.5-fold higher levels of IFN activity were observed in IRF-3^−/−^ mice at 48 h after infection (*p* < 0.0001). This data is consistent with previous studies with EMCV and HSV [22] in which a deficiency of IRF-3 did not significantly diminish systemic accumulation of IFN.

### Effect of IRF-3 on WNV Infection and IFN Induction in Macrophages

Although the IRF-3^−/−^ mice showed significant virologic and mortality phenotypes, small differences were observed in IFN-α and IFN-β gene induction and antiviral activity in the lymph node and intravascular compartments. Because IRF-3 can also directly induce IFN-stimulated antiviral genes in an IFN-independent manner [29–31], we hypothesized that some of its inhibitory functions against WNV could be direct and independent of IFN. To address this question, we isolated resting macrophages from wild-type and IRF-3^−/−^ mice and performed virologic and protein expression analyses. Macrophages were chosen for study because subsets of these cells are infected in vivo by WNV [4].

**Figure 3:**
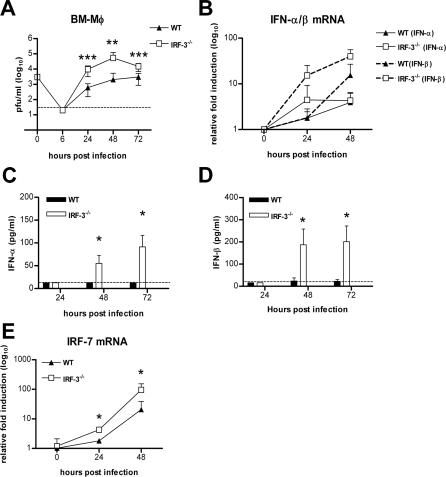
IRF-3 Modulates WNV Infection in Primary Macrophages (A) Macrophages generated from wild-type or IRF-3^−/−^ mice were infected at an MOI of 0.01, and virus production was evaluated at the indicated times post infection by plaque assay. Values are an average of quadruplicate samples generated from at least three independent experiments. Error bars represent the SD, and asterisks indicate differences that are statistically significant relative to wild-type mice (*, *p* < 0.05; **, *p* < 0.005; ***, *p* < 0.0001). (B) The induction of IFN-α and IFN-β mRNA in WNV-infected macrophages was analyzed by qRT-PCR as described in Figure 2. (C, D) Accumulation of IFN-α (C) and IFN-β (D) protein in supernatants of WNV-infected macrophages was determined by ELISA. The data is the average of at least five independent experiments performed in triplicate. *, *p* < 0.05. (E) The induction of IRF-7 mRNA in WNV-infected macrophages was analyzed by qRT-PCR as described in Figure 2. *, *p* < 0.05.

Increased WNV replication was observed in IRF-3^−/−^ macrophages beginning at 24 h after infection (Figure 3). An absence of IRF-3 resulted in a 16-fold, 26-fold, and 5-fold increase in viral titers at 24, 48, and 72 h, respectively (*p* < 0.002; Figure 3A). To determine whether the increased infection in IRF-3^−/−^ macrophages was due to altered IFN levels, we analyzed the kinetics of IFN-α and IFN-β production in WNV-infected wild-type and IRF-3^−/−^ cells. Paradoxically, earlier and higher levels of IFN-α and IFN-β mRNA induction were observed in WNV-infected IRF-3^−/−^ macrophages (Figure 3B). Consistent with this, at 48 and 72 h, higher levels of IFN-α and IFN-β were observed in supernatants from WNV-infected IRF-3^−/−^ cells (Figure 3C and 3D). We hypothesize that this increased IFN production is secondary to the significantly increased viral infection in IRF-3^−/−^ cells. To determine whether other transcriptional regulators that induce IFN gene expression could be compensating for a deficiency of IRF-3, we analyzed induction of IRF-5 and IRF-7 after WNV infection. Notably, IRF-7 levels were ∼2.5 and ∼5-fold higher (*p* < 0.05; Figure 3E) in IRF-3^−/−^ macrophages at 24 and 48 h after WNV infection, respectively. In contrast, no difference in IRF-5 levels was observed (unpublished data). Thus, in primary macrophages, induction of IFN-α and IFN-β after WNV infection is IRF-3-independent and likely IRF-7-dependent. Moreover, in these cells, IRF-3 restricts WNV replication independently of IFN.

**Figure 4:**
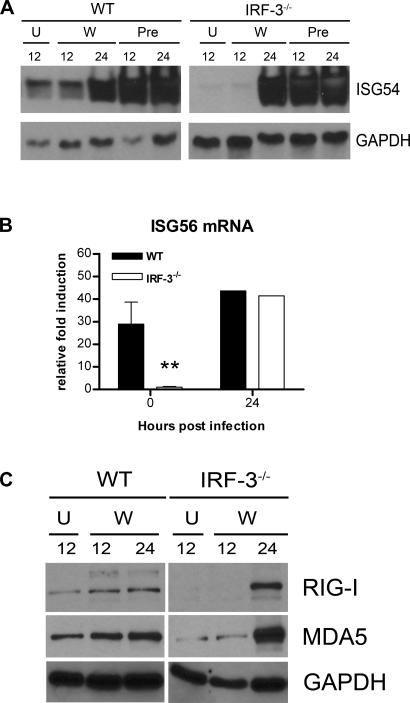
The Effect of IRF-3 on ISG54, ISG56, RIG-I, and MDA5 Expression in Macrophages (A) Whole cell lysates were generated at the indicated times from wild-type or IRF-3^−/−^ macrophages that were uninfected (U), infected with WNV (W), or pretreated with 100 IU/ml of IFN-β (Pre). Protein levels of ISG54 and GAPDH were examined by immunoblot analysis. (B) Total RNA was harvested from WNV-infected macrophages and mRNA levels of ISG56 were quantified by qRT-PCR as described in Figure 2. Data are expressed as relative fold induction over uninfected IRF-3^−/−^ cells. Asterisks indicate differences that are statistically significant (**, *p* < 0.005). (C) Whole cell lysates were generated at the indicated times from wild-type or IRF-3^−/−^ macrophages that were uninfected (U) or infected with WNV (W) for 12 or 24 h. Protein levels of RIG-I, MDA5, and GAPDH were examined by immunoblot analysis

As IRF-3 did not modulate IFN production during WNV infection, we evaluated its role in directly inducing expression of ISGs; some ISGs have antiviral activity and could contribute to differences in viral growth curves. ISG54 and ISG56 are IRF-3 target genes that inhibit translation [29,30,32] and are up-regulated in WNV-infected MEFs [14]. Thus, we assessed the expression of ISG54 by Western blot and ISG56 by qRT-PCR in macrophages (Figure 4A and 4B). Basal expression of ISG54 and ISG56 was observed in wild-type but not in IRF-3^−/−^ cells. However, infection with WNV or pretreatment with IFN-β (100 IU/ml) induced ISG54 and ISG56 expression in both wild-type and IRF-3^−/−^ cells (Figure 4A and unpublished data). Thus, ISG54 and ISG56 are basally expressed in primary macrophages in an IRF-3-dependent manner, yet induced through an IRF-3-independent and IFN-dependent pathway.

Since the basal levels of ISG54 and ISG56 were lower in IRF-3^−/−^ compared to wild-type macrophages, we speculated that IRF-3 might also indirectly affect the levels of the cytosolic double-stranded viral RNA sensors (RIG-I and MDA5). Basal expression of RIG-I and MDA5 was present in wild-type macrophages, but noticeably lower in IRF-3^−/−^ cells (Figure 4C). After WNV infection, RIG-I and MDA5 were induced in an IRF-3-independent manner in macrophages. Overall, these experiments suggest that macrophages, because of their IRF-3-dependent basal expression of RIG-I, MDA5, and specific ISG, are poised to detect cytoplasmic viral RNA and generate an early host response that limits viral replication.

### Effect of IRF-3 on the Control of Neuronal Infection

Mice deficient in IRF-3 showed increased WNV burden in the brain and spinal cord after peripheral infection. This phenotype could be due either to increased dissemination from the periphery and/or an independent antiviral effect in the CNS. To test this, wild-type and IRF-3^−/−^ mice were infected with 10^1^ PFU of WNV via an intracranial (IC) route and survival was monitored (Table 1). Both groups of mice showed rapid and complete mortality following IC infection, though IRF-3^−/−^ mice showed a slight but significantly decreased average survival time relative to wild-type mice (mean time to death of 6.5 and 7.2 for the IRF-3^−/−^ and the wild-type mice, respectively, *p* = 0.003). These data suggest that IRF-3 has a subtle yet independent function in protection against WNV in the CNS. To further examine the role of IRF-3 in the CNS, we measured viral burden in the brain and spinal cord on days 4 and 6 after IC inoculation. IRF-3^−/−^ animals had significantly higher mean viral burdens at day 4 after infection in brain (10^7.6^ PFU/g for IRF-3^−/−^ and 10^5.7^ PFU/g for wild-type, *p* = 0.02) and spinal cord (10^5.7^ PFU/g for IRF-3^−/−^ and 10^4.0^ PFU/g for wild-type, *p* = 0.008). By day 6 after infection, no significant differences in viral titers were observed between wild-type and IRF-3^−/−^ mice (*p* > 0.3 and *p* > 0.7 in brain and spinal cord, respectively).

**Table 1:**
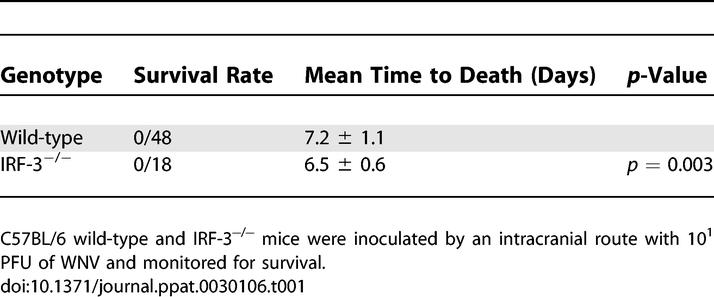
Survival of Wild-Type and IRF-3^−/−^ C57BL/6 Mice following IC Inoculation

### Effect of IRF-3 on WNV Infection and IFN Induction in Primary Cortical Neurons

Production of type I IFN by neurons has been observed after infection of other RNA viruses and could serve an antiviral role in vivo [33,34]. To evaluate whether IRF-3 directly modulates neuronal infection or IFN production, we generated primary cortical neuron cultures [35,36] from wild-type and IRF-3^−/−^ mice. Neurons in the cerebral cortex are targets of WNV infection [6], can be generated with high purity (∼98%–99%), and are acutely susceptible to WNV infection in vitro [36].

**Figure 5:**
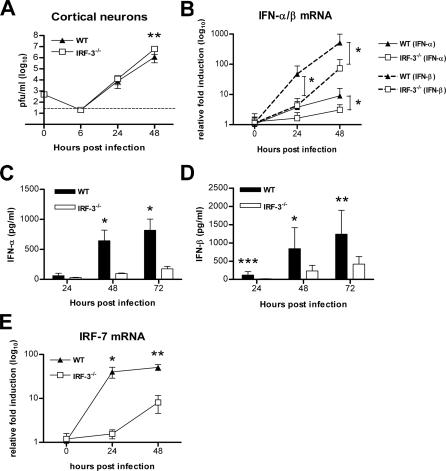
WNV Infection and IFN Production in Primary Cortical Neurons (A) Primary cortical neurons generated from wild-type or IRF-3^−/−^ mice were infected at an MOI of 0.001, and virus production was evaluated at the indicated times by plaque assay. Values are an average of triplicate samples generated from three independent experiments, error bars represent the SD, and asterisks indicate values that are statistically significant (**, *p* < 0.005). (B–D) The induction of IFN-α and IFN-β mRNA in WNV-infected primary cortical neurons. IFN mRNA was analyzed by qRT-PCR as described in Figure 2. (C) IFN-α and (D) IFN-β protein accumulation in supernatants of WNV-infected cortical neurons from wild-type and IRF-3^−/−^ mice was measured by ELISA. (E) The induction of IRF-7 mRNA in WNV-infected primary cortical neurons was analyzed by qRT-PCR as described in Figure 2. (B–E) Data are the average of three independent experiments performed in duplicate, and the asterisks indicate statistically significant differences (*, *p* < 0.05; **, *p* < 0.005, ***, *p* < 0.0001).

Wild-type neurons were significantly more permissive to WNV infection than macrophages: infection with 10-fold less virus (multiplicity of infection [MOI] 0.001 versus 0.01) produced ∼560-fold more virus within 48 h (*p* < 0.0001) (Figures 3A and Figure 5A). Multi-step growth curve analysis of wild-type and IRF-3^−/−^ cortical neurons showed no difference at 24 h and only a small (5-fold, *p* = 0.005) increase in WNV infection in the absence of IRF-3 at 48 h (Figure 5A). Unlike macrophages, cortical neurons required IRF-3 for efficient IFN production, as decreased levels of IFN-α (at 48 h, 6.8-fold, *p* = 0.02; 72 h, 4.7-fold, *p* = 0.02) and IFN-β (24 h, 7.5-fold, *p* = 0.001; 48 h, 3.6-fold, *p* = 0.04; and 72 h, 2.9-fold, *p* = 0.009) were detected in the supernatants of WNV-infected IRF-3^−/−^ neurons (Figure 5C and 5D). Kinetic analysis of IFN gene induction also showed markedly higher (12- to 55-fold) IFN-β levels compared to IFN-α levels at 24 to 48 h after infection in wild-type neurons and blunted IFN transcript levels in WNV-infected IRF-3^−/−^ neurons (Figure 5B). As IFN pretreatment inhibits WNV infection in cortical neurons a maximum of 5- to 8-fold [4], the modest viral replication phenotype in IRF-3^−/−^ neurons is consistent with the decreased IFN production by these cells. To determine whether the IFN phenotype in IRF-3^−/−^ neurons was associated with altered expression of other IFN transcriptional regulators, we analyzed the induction of IRF-7. Notably, IRF-7 mRNA levels were ∼25- and ∼5-fold lower (*p* < 0.05; Figure 5E) in IRF-3^−/−^ neurons at 24 and 48 h after WNV infection, respectively. Thus, the defect of IFN-production in IRF-3^−/−^ cortical neurons may occur because of blunted IRF-7 induction.

**Figure 6:**
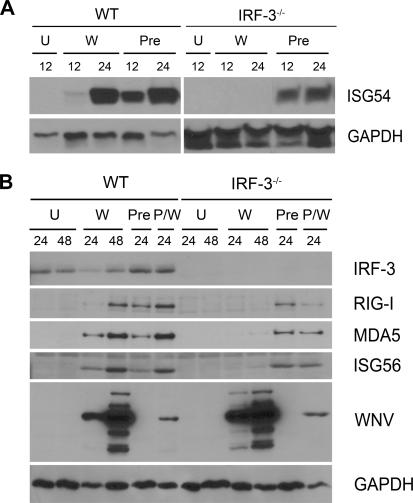
The Effect of IRF-3 on ISG54, ISG56, RIG-I, and MDA5 Expression in WNV-Infected Cortical Neurons (A, B) Whole cell lysates were generated at the indicated times from wild-type or IRF-3^−/−^ primary cortical neurons. These neurons were uninfected (U), infected with WNV (W), pretreated with 100 IU/ml of IFN-β (Pre), or treated with IFN after infection (P/W). (A) ISG54 immunoblot analysis and figure labeling is as described in Figure 4. (B) Protein levels of IRF-3, RIG-I, MDA5, ISG56, WNV, and GAPDH were examined by immunoblot analysis as described in Materials and Methods.

To further explore the function of IRF-3 in antiviral signaling pathways in cortical neurons, we assessed the levels of ISG54 and ISG56 (Figure 6A and 6B). Unlike macrophages, ISG54 and ISG56 were not expressed basally in uninfected wild-type cortical neurons. Also, in contrast to macrophages, a deficiency of IRF-3 abolished production of ISG54 and ISG56 in neurons after WNV infection. Nonetheless, pretreatment with IFN-β (100 IU/ml) strongly induced ISG54 and ISG56 in wild-type and IRF-3^−/−^ neurons. Taken together, these results suggest that IRF-3 is required in cortical neurons both for optimal induction of antiviral ISGs and IFN production after WNV infection. Surprisingly, RIG-I and MDA-5 were not basally expressed in wild-type or IRF-3^−/−^ cortical neurons but were induced in an IRF-3-dependent fashion at 24 and 48 h after infection (Figure 6B). Thus, in cortical neurons, the intracellular viral RNA sensors RIG-I and MDA5 are absent when WNV enters the cytoplasm but are induced after infection through an IRF-3-dependent mechanism.

## Discussion

An intact type I IFN response controls WNV infection in mice and limits replication in specific cell populations [9]. In vitro studies in MEFs suggested that the host response restricts WNV spread, in part, through activation of the IRF-3 pathway [14]. In this study, we established IRF-3 as an essential regulator of the early host response in vivo after WNV infection. Mice lacking IRF-3 were uniformly vulnerable with increased viremia, higher viral burden in peripheral tissues, altered tissue tropism, and earlier entry into the CNS. Ex vivo studies showed that specific primary cell types utilize IRF-3 with distinct functional consequences: in macrophages, IRF-3 was required for basal ISG expression and control of WNV infection, whereas in neurons, IRF-3 was necessary for efficient IFN induction. Remarkably, in vitro studies in myeloid cells and in vivo experiments suggested that a deficiency of IRF-3 did not dramatically diminish the peripheral IFN response to WNV infection, yet was still required for early control of WNV replication and spread. Thus, IRF-3 plays distinct roles in diverse cell types to limit WNV through IFN-dependent and -independent mechanisms.

Host pathogen recognition receptors sense RNA viruses and direct an early host response through IRF activation and the induction of IFN-β and ISGs. For example, RIG-I, MDA5, and TLR3 all recognize double-stranded viral RNA and signal through IRF-3 [15,26,27]. Although IRF-3 plays a key role in limiting WNV infection in vitro in MEFs [14], its function in triggering host defense responses after WNV infection in vivo in cell types relevant to pathogenesis was unknown. Our experiments demonstrate that IRF-3 is absolutely required for the immediate control of WNV replication in vivo: increased viral burden and uniform death were observed in IRF-3^−/−^ mice within 10 d of peripheral WNV infection. The increased level of WNV in IRF-3^−/−^ mice did not correlate with significant effects on systemic production of IFN-α and IFN-β; these results are consistent with studies of EMCV, HSV, and SFV infection of IRF-3^−/−^ mice [22,23]. This suggests that the protective effect of IRF-3 is largely independent of the production of IFN-α and IFN-β in the periphery, and instead is due likely to the antiviral actions of specific IRF-3 target genes. However, similar to IFN-α/βR^−/−^ mice [9], WNV infection of IRF-3^−/−^ mice also resulted in altered tissue tropism. Analysis of the cellular basis of expanded tropism in IFN-α/βR^−/−^ mice showed increased infection of macrophages [9]. As a deficiency of IRF-3 in macrophages ex vivo significantly altered basal expression of key host defense molecules, the expanded in vivo tropism in IRF-3^−/−^ mice may be due, in part, to enhanced infection in normally resistant tissue macrophages.

Although it is not known which pathogen recognition receptor contributes to the recognition of WNV in vivo, RIG-I is a likely candidate. TLR3^−/−^ mice showed only moderately increased WNV infection in peripheral tissues [17], and MDA5^−/−^ mice showed no difference in susceptibility compared to wild-type mice with the closely (∼90% amino acid identity) related flavivirus, Japanese encephalitis virus. In contrast, RIG-I^−/−^ mice were highly vulnerable to Japanese encephalitis virus infection, with 100% mortality after 7 d [15]. Further studies are required to evaluate the relative role of different pathogen recognition receptors in sensing WNV and inducing an antiviral response.

IRF-3 appeared to exert its antiviral activity by modulating the antiviral state of individual cell types. Our results in IRF-3^−/−^ macrophages agree with studies of IRF-3^−/−^ splenic dendritic cells, which showed relatively normal IFN induction after infection with VSV or HSV [22]. However, they contrast with studies of MEFs or myeloid dendritic cells whereby IRF-3 was required to fully activate IFN-α and IFN-β responses after infection with HSV, VSV, EMCV [19,22], or SFV [23]. These latter results are more consistent with our cortical neuron data, in which induction of IFN-α and IFN-β was regulated, in part, by an IRF-3-dependent mechanism. We propose a model in which the activation of IFN genes after virus recognition in different cells is mediated by IRF-3-dependent and -independent pathways. Although IRF-7 is an obvious candidate for the IRF-3-independent pathway, [37], other IRF family members could regulate IFN gene expression. IRF-5 and IRF-8 have been implicated in IFN gene induction in macrophages and dendritic cells after Newcastle disease virus and Sendai virus infection [38–40]. Our preliminary data suggest that IRF-7 may have a dominant role in IFN induction: IRF-3^−/−^ macrophages that had no defect in IFN production showed strong induction of IRF-7 mRNA after WNV infection. In contrast, IRF-3^−/−^ cortical neurons, which had blunted production of IFN, also had markedly lower levels of IRF-7 mRNA after infection. Clearly, additional studies are required to establish the precise cell-specific regulatory pathways for IFN induction after WNV and other viral infections.

IRF-3 has been previously shown to contribute to an antiviral state by activating ISGs, including ISG54 and ISG56, in an IFN-dependent and -independent manner in fibroblasts and epithelial cells infected with HSV, VSV, Newcastle disease, vaccinia, and Sendai viruses [31]. When we examined the expression of ISG54 and ISG56, genes that are directly regulated by IRF-3 [30], activated after WNV infection [14], and have antiviral activity [29], we found that ISG54 and ISG56 were basally expressed in macrophages in an IRF-3-dependent manner. Similar findings were observed for the intracellular double-stranded RNA sensors MDA5 and RIG-I. However, after WNV infection or pretreatment with IFN-β, ISG54, ISG56, and RIG-I induction occurred independently of IRF-3. Thus, the levels of these host defense genes in myeloid cells are modulated both by IRF-3 and IFN-α and IFN-β. We speculate that the relatively low permissiveness of wild-type but not IRF-3^−/−^ macrophages could be due to basal expression of ISGs (ISG54, ISG56, and/or other IRF-3 target ISGs) and pathogen recognition molecules (RIG-I and MDA5).

Although IRF-3 had a significant and yet largely IFN-independent antiviral effect in myeloid cells and peripheral tissues, it had a distinct function in neurons. IRF-3^−/−^ mice developed higher CNS viral burden more rapidly after IC inoculation, and the WNV-induced IFN production in IRF-3^−/−^ cortical neurons was impaired. Despite reduced expression of several (ISG54, ISG56, RIG-I, and MDA5) host defense molecules and lower IFN production, the absence of IRF-3 had a smaller effect on WNV replication in neurons. The absence of basal RIG-I, MDA5, and ISG expression and less efficient IFN antiviral effector pathways could contribute to high baseline permissiveness of cortical neurons for WNV and perhaps other neurotropic RNA viruses. Indeed, pretreatment of myeloid cells with IFN-β reduced viral titer by 1,000- to 10,000-fold, but inhibited WNV infection in cortical neurons by only 5- to 8-fold [4]. So, if IRF-3 does not dramatically affect WNV infection in cortical neurons, why is there increased replication in the CNS of IRF-3^−/−^ mice? We speculate that IRF-3-dependent production of IFN-α and IFN-β in infected cortical neurons has a paracrine antiviral effect on other neuronal and non-neuronal (e.g., astrocytes, oligodendrocytes, and microglia) cells in the CNS.

In summary, our experiments suggest that IRF-3 signals the host to control viral infections and/or initiate IFN responses by distinct mechanisms in different cell types. The differential ability to signal through IRF-3 reflects cell-specific expression of pathogen recognition receptors, which alters the baseline antiviral state of the cell and the capacity to induce IFN responses. IRF-3-dependent basal expression of pathogen recognition receptors and selected ISGs in specific cell types likely expedites the induction of IFN and possibly other cell-specific antiviral effector molecules. To this end, genetic profiling studies are underway with wild-type and deficient myeloid and neuronal cells to identify novel cell-specific antiviral pathways that inhibit WNV and other viral infections.

## Materials and Methods

### Mouse experiments and quantitation of viral burden.

C57BL/6 wild-type inbred mice were commercially obtained (Jackson Laboratory, http://www.jax.org/). The congenic, backcrossed IRF-3^−/−^ mice have been previously published [19] and were the generous gift of T. Taniguchi (Tokyo, Japan). All mice were genotyped and bred in the animal facilities of the Washington University School of Medicine, and experiments were performed in accordance with Washington University animal studies guidelines. Eight- to twelve-week-old mice were used for all in vivo studies. For peripheral infection, 10^2^ PFU of WNV was diluted in Hanks balanced salt solution (HBSS) supplemented with 1% heat-inactivated fetal bovine serum (FBS) and inoculated by footpad injection in a volume of 50 μl. IC inoculation was performed by injecting 10^1^ PFU of WNV diluted in 10 μl of HBSS with 1% FBS.

### Viruses.

The WNV strain (3000.0259) was isolated in New York in 2000 [41] and passaged once in C6/36 cells to generate a stock virus that was used in all experiments.

### Quantification of tissue viral burden and viremia.

To monitor viral spread in vivo, mice were infected with 10^2^ PFU of WNV by footpad inoculation and euthanized at days 1, 2, 4, 6, 8, and 10 after inoculation. In some experiments, mice were infected with 10^1^ PFU of WNV by an intracranial route and euthanized at days 4 and 6 after infection. After extensive cardiac perfusion with PBS, organs were harvested, weighed, and homogenized, and virus was titrated by standard plaque assay as previously described [42]. Viral burden also was measured by analyzing RNA levels using fluorogenic qRT-PCR as previously described [4].

### Detection of IFN activity in serum using an L929 cell bioassay.

Biologically active IFN was detected and quantified using an EMCV L929 cytopathic effect bioassay as described previously [4]. The percentage of protected cells was calculated as described [43], according to the following formula: (optical density at 492 nm [OD_492_] of mouse serum-treated EMCV-infected cells/ OD_492_ of non-EMCV-infected cells − OD_492_ of EMCV-infected cells/ OD_492_ of non-EMCV-infected cells) × 100%).

### Measurement of WNV-specific antibodies.

The levels of WNV-specific IgM and IgG were determined using an ELISA against purified WNV E protein as described previously [44].

### Quantification of mRNA levels by qRT-PCR.

Total RNA was isolated from lymph nodes or primary cells by using the RNeasy kit according to the manufacturer's instructions (Qiagen, http://www.qiagen.com/). During the isolation, to remove any contaminating DNA, samples were treated with RNAse-free DNAse (Qiagen). mRNA were amplified and quantified from total RNA by qRT-PCR as previously described [9]. The following primers and probes were used to amplify murine IFN-α, IFN-β, ISG56, and IRF-7 mRNA: IFN-α, forward primer, 5′-CTTCCACAGGATCACTGTGTACCT-3′, reverse primer, 5′TTCTGCTCTGACCACCTCCC3′, probe, 5′-FAM-AGAGAGAAGAAACACAGCCCCTGTGCC-TAMRA-3′; IFN-β, forward primer, 5′-CTGGAGCAGCTGAATGGAAAG-3′, reverse primer, 5′-CTTCTCCGTCATCTCCATAGGG-3′, probe 5′-FAM-CAACCTCACCTACAGGGCGGACTTCAAG-TAMRA-3′; ISG56, forward primer, 5′-GAGCCAGAAAACCCTGAGTACA-3′, reverse primer, 5′-AGAAATAAAGTTGTCATCTAAATC-3′, probe 5′-FAM-ACTGGCTATGCAGTCGTAGCCTATCGCC-TAMRA-3′; and IRF-7, forward primer, 5′-CTGGAGCCATGGGTATGCA-3′, reverse primer, 5′-AAGCACAAGCCGAGACTGCT-3′, probe 5′-FAM-CTGGAGGGCGTGCAGCGTGA-TAMRA-3′. To analyze the relative fold induction of amplified mRNA, 18S rRNA expression levels were also determined for normalization by using the Ct method as described previously [45].

### Macrophage infection.

Bone marrow–derived macrophages were generated as described previously [4]. Briefly, cells were isolated from the bone marrow of wild-type or IRF-3^−/−^ mice and cultured for 7 d in the presence of 40 ng/ml M-CSF (PeproTech, http://www.peprotech.com/) to generate macrophages. Multi-step virus growth curves were performed after infection at an MOI of 0.01. Supernatants were titrated by plaque assay on BHK21–15 cells. To test for induction of IFN-α and IFN-β and IRF-7 genes after WNV infection, 5 × 10^5^ macrophages were infected at an MOI of 0.1. IFN mRNA was measured by qRT-PCR as described above. To measure secreted IFN-α and IFN-β protein levels in macrophage supernatants, a commercial capture ELISA was used according to the manufacturer's instructions (PBL Biomedical Laboratories, http://www.interferonsource.com/).

### Neuron infection.

Primary cortical neurons were prepared from day 15 wild-type and IRF-3^−/−^ mouse embryos as previously described [35,36]. Neurons were seeded in 24-well poly-D-lysine/laminin-coated plates in Dulbecco's modified Eagle's medium (DMEM) containing 5% heat-inactivated FBS and 5% horse serum for 24 h. Cortical neurons were then cultured for 4 d with Neurobasal medium containing B27 and L-Glutamine (Invitrogen, http://www.invitrogen.com/). Multi-step virus growth curves and IFN-α and IFN-β and IRF-7 gene induction and protein secretion assays were performed after infection at an MOI of 0.001 and 0.1, respectively.

### Western blots.

Macrophages (10^6^) or cortical neurons (10^6^) were lysed in RIPA buffer (10 mM Tris, 150 mM NaCl, 0.02% sodium azide, 1% sodium deoxycholate, 1% Triton X-100, 0.1% SDS [pH 7.4]), with protease inhibitors (Sigma, http://www.sigmaaldrich.com/) and 1 mM okadaic acid (Sigma). Samples (30 μg) were resolved on 10% SDS-polyacrylamide gels. Following transfer, membranes were blocked with 5% nonfat dried milk overnight at 4 °C. Membranes were probed with the following panel of monoclonal or polyclonal antibodies: anti-WNV (US Centers for Disease Control and Prevention, Atlanta, Georgia, United States), anti-GAPDH (Santa Cruz Biotechnology, http://www.scbt.com/), anti-mouse IRF-3 (Invitrogen), anti-mouse MDA5 (Axxora, http://www.axxora.com/), and anti-mouse ISG56 and anti-mouse ISG54 (gifts from G. Sen, Cleveland, Ohio, United States). The RIG-I antibody was raised in rabbits using a recombinant N-terminal fragment of RIG-I (amino acids 1–228) that was expressed in Escherichia coli and purified by size exclusion chromatography. Blots were incubated with peroxidase-conjugated secondary antibodies (Jackson ImmunoResearch, http://www.jacksonimmuno.com/) and visualized using ECL-Plus Immunoblotting reagents (Amersham Biosciences, http://www.gelifesciences.com/).

### Statistical analysis.

For in vitro experiments, an unpaired *t*-test was used to determine statistically significant differences. For viral burden analysis, differences in log titers were analyzed by the Mann–Whitney test. Kaplan–Meier survival curves were analyzed by the log rank test. All data were analyzed using GraphPad Prism 4 software (GraphPad Software, http://www.graphpad.com/).

## Abbreviations

CNS: central nervous system
EMCV: encephalomyocarditis virus
HSV: herpes simplex virus
IC: intracranial
IFN: interferon
IRF: interferon regulatory factor
ISG: interferon-stimulated gene
MDA: melanoma differentiation antigen
MEF: murine embryonic fibroblast
MOI: multiplicity of infection
qRT-PCR: quantitative reverse transcriptase polymerase chain reaction
RIG: retinoic acid–inducible gene
SFV: Semliki Forest virus
TLR: Toll-like receptor
VSV: vesicular stomatitis virus
WNV: West Nile virus
